# An Eastern Antidote against Fever

**Published:** 1865-10

**Authors:** 


AN EASTERN ANTIDOTE AGAINST FEVER.—There is a great mortality from a description of fever, just now, in Gwalior. The Maharajah has ordered the perpetual performance of various religious ceremonies, with a view of checking the course of the epidemic.

